# Dynamic changes of amplitude of low‐frequency fluctuations in patients with generalized anxiety disorder

**DOI:** 10.1002/hbm.24902

**Published:** 2019-12-17

**Authors:** Qian Cui, Wei Sheng, Yuyan Chen, Yajing Pang, Fengmei Lu, Qin Tang, Shaoqiang Han, Qian Shen, Yifeng Wang, Ailing Xie, Jing Huang, Di Li, Ting Lei, Zongling He, Huafu Chen

**Affiliations:** ^1^ School of Public Affairs and Administration University of Electronic Science and Technology of China Chengdu China; ^2^ The Clinical Hospital of Chengdu Brain Science Institute, MOE Key Lab for Neuroinformation, School of Life Science and Technology University of Electronic Science and Technology of China Chengdu China; ^3^ Education Center for Students Cultural Qualities University of Electronic Science and Technology of China Chengdu China

**Keywords:** dynamic amplitude of low‐frequency fluctuations, generalized anxiety disorder, local brain activity, resting‐state fMRI, variability

## Abstract

Previous neuroimaging studies have mainly focused on alterations of static and dynamic functional connectivity in patients with generalized anxiety disorder (GAD). However, the characteristics of local brain activity over time in GAD are poorly understood. This study aimed to investigate the abnormal time‐varying local brain activity of GAD by using the amplitude of low‐frequency fluctuation (ALFF) method combined with sliding‐window approach. Group comparison results showed that compared with healthy controls (HCs), patients with GAD exhibited increased dynamic ALFF (dALFF) variability in widespread regions, including the bilateral dorsomedial prefrontal cortex, hippocampus, thalamus, striatum; and left orbital frontal gyrus, inferior parietal lobule, temporal pole, inferior temporal gyrus, and fusiform gyrus. The abnormal dALFF could be used to distinguish between patients with GAD and HCs. Increased dALFF variability values in the striatum were positively correlated with GAD symptom severity. These findings suggest that GAD patients are associated with abnormal temporal variability of local brain activity in regions implicated in executive, emotional, and social function. This study provides insight into the brain dysfunction of GAD from the perspective of dynamic local brain activity, highlighting the important role of dALFF variability in understanding neurophysiological mechanisms and potentially informing the diagnosis of GAD.

## 1 INTRODUCTION

Generalized anxiety disorder (GAD) is a prevalent mental disorder characterized by inexplicable, chronic, and persistent worrying (Tyrer & Baldwin, 2006) Patients with GAD frequently worry about ongoing things in their daily life or potential future outcomes (Li, Duan, Cui, Chen, & Liao, 2019). Most patients often suffer from a series of physical or psychological somatic symptoms, such as restlessness, fatigue, difficulty concentrating, irritability, and sleep disturbances (American Psychiatric Association, 2013). Considering the high morbidity, severe distress (Molent et al., 2018), high financial burden, and low remission rate after treatment compared to those for other anxiety disorders (Christine Buff et al., 2016; Kinney, Boffa, & Amir, 2017), the pathological mechanisms underlying GAD should be elucidated to facilitate more effective therapeutic development.

Previous neuroimaging studies have revealed brain functional abnormalities in patients with GAD, which were frequently characterized by task and resting‐state functional magnetic resonance imaging (fMRI) findings. Task‐related fMRI studies have reported hypoactivation of the prefrontal cortex (PFC), anterior cingulate cortex (ACC) (Palm, Elliott, McKie, Deakin, & Anderson, 2011; Wang et al., 2018), parietal cortex, and fusiform (Moon & Jeong, 2015; Wang et al., 2018), as well as hyperactivation of the amygdala (Fonzo et al., 2015; McClure et al., 2007; Park, Kim, Jeong, Chung, & Yang, 2016) and hippocampus (Moon & Jeong, 2015; Park et al., 2016) in GAD patients when confronting emotion‐inducing stimuli, especially processing stimuli with negative emotional valence. Consistent with these findings, resting‐state fMRI studies have demonstrated that patients with GAD show widespread abnormal resting‐state functional connectivity (rsFC) involving regions of the prefrontoparietal cognitive control network such as the PFC, ACC, and parietal cortex (Andreescu, Sheu, Tudorascu, Walker, & Aizenstein, 2014; Etkin, Prater, Schatzberg, Menon, & Greicius, 2009; Roy et al., 2013); limbic regions including the amygdala, hippocampus, thalamus, and insula (Chen & Etkin, 2013; Cui et al., 2016; Etkin et al., 2009; Makovac et al., 2016; Qiao et al., 2017; Roy et al., 2013); and regions associated with socioemotional processing such as the striatum, fusiform gyrus, and subregions of the temporal lobe (Cui et al., 2016; Etkin et al., 2009; Qiao et al., 2017). Most of these studies rely on the implicit assumption that brain activity remains stationary during fMRI scanning. However, an increasing number of recent studies propose that brain activity is dynamic over time (Allen et al., 2014; Hutchison et al., 2013; Li et al., 2018).

Dynamic characteristics of brain activity have been associated with cognitive adaption (Fornito, Harrison, Zalesky, & Simons, 2012; Wang, Ong, Patanaik, Zhou, & Chee, 2016), brain development (Faghiri, Stephen, Wang, Wilson, & Calhoun, 2018), and mental disorders (Li, Liao, et al., 2018; Liao et al., 2018; Zhang et al., 2018), which have been investigated in several neurological and psychiatric disorders, including depression (Pang et al., 2018), autism (Guo et al., 2018), schizophrenia (Damaraju et al., 2014), and GAD (Li et al., 2019; Yao et al., 2017). These studies demonstrate the utility of brain dynamics in deepening our understanding of the diseased brain and highlight its potential role in improving diagnostic accuracy. As recently reported, dynamic functional connectivity (dFC) can be used to distinguish patients with GAD from healthy controls (HCs) and exhibits high accuracy (Yao et al., 2017). However, most of these studies have focused on reoccurring patterns of connection among brain regions using the dFC method. To date, the dynamic characteristics of local brain activity have rarely been investigated. Local brain activity reflects aspects of the intrinsic property of brain fluctuation organization (Ralchle & Snyder, 2007) and associated with mental and cognitive processes (Britz, Pitts, & Michel, 2011; Hutchison & Morton, 2016). The amplitude of low‐frequency fluctuations (ALFF) is an effective approach to measure local brain activity (Zang et al., 2007). Combining the ALFF with “sliding‐window” approaches, the dynamic ALFF (dALFF) method was proposed to measure the variance of ALFF over time. The dALFF provides a new avenue to depict time‐varying local brain activity (Liao et al., 2019) and has been applied in patients with depression (Li, Duan, et al., 2019) and schizophrenia (Fu et al., 2018; Li, Duan, et al., 2019) reported that abnormal patterns of dALFF variability in depression could be used to predict patients' suicidal ideation (Li, Duan, et al., 2019). However, whether patients with GAD exhibit abnormal dynamic local brain activity remains unclear. Identifying such abnormalities would promote our understanding of the neuropathological mechanisms underlying GAD.

In the present study, we explored the dynamic local brain activity in patients with GAD using the ALFF method combined with sliding‐window approach. We expected that patients with GAD would show altered dALFF patterns compared to those of HCs, and that such abnormalities may underline clinical symptomatology in GAD and could be used as features to distinguish patients with GAD from HCs.

## 2 METHODS

### 2.1 Participants

In total, 56 patients with GAD were recruited from the Clinical Hospital of Chengdu Brain Science Institute, University of Electronic Science and Technology of China, and 55 HCs were recruited from the local community through advertisements. All patients were interviewed by two experienced psychiatrists and met the diagnostic criteria for GAD as defined by the Structured Clinical Interview for DSM‐IV (SCID‐IV patient edition). The clinical states of patients were assessed using the 14‐item Hamilton Anxiety Rating Scale (HAMA). Exclusion criteria included major depressive disorder, obsessive–compulsive disorder, post‐traumatic stress disorder, substance or alcohol abuse disorder, and any history of head trauma or unconsciousness. We excluded patients with comorbid anxiety and depression to reduce the heterogeneity of patients, since GAD and GAD with comorbid depression have been reported to have different neuropathological mechanisms (O'Garro‐Moore, Adams, Abramson, & Alloy, 2015; Tully & Cosh, 2013). In total, 45 patients received medication treatment, of which selective serotonin reuptake inhibitors were prescribed for 37 patients, including fluoxetine (*n* = 3), sertraline (*n* = 6), paroxetine (*n* = 15), citalopram (*n* = 1), escitalopram (*n* = 11), and fluvoxamine (*n* = 1); serotonin and norepinephrine reuptake inhibitors were prescribed for eight patients, including venlafaxine (*n* = 4) and duloxetine (*n* = 4). *N* indicates the number of patients. The HC group was screened using the SCID nonpatient edition. The two groups were matched for age, sex, years of education, handedness, and mean framewise displacement (FD) for head motion (see FD calculation in Section 2.3). The detailed clinical and demographic data of the two groups are listed in Table 1. All subjects were provided information about the procedure and aims of the study, and provided their written informed consent before experimentation. This study was approved by the ethical committee of the University of Electronic Science and Technology of China and registered at http://clinicaltrials.gov (Identifier: NCT02888509).

**Table 1:** Characteristics of demographic and clinical variables of HC and patients with GAD

Variables	HC (*n* = 55)	GAD (*n* = 56)	Statistics	*p*‐Value
Age (years)	32.98 ± 11.06	35.39 ± 8.67	1.28	.20^a^
Sex (male/female)	25/30	20/36	—	.30^b^
Handedness (left/right)	1/54	4/52	—	.18^b^
Education (years)	13.29 ± 3.75	12.06 ± 3.37	1.86	.07^a^
Mean FD	0.10 ± 0.05	0.10 ± 0.07	0.12	.90^a^
Duration of illness (months)	—	51.16 ± 64.68	—	—
Age of first onset (years)	—	31.14 ± 9.46	—	—
No. anxiety episodes	—	2.16 ± 1.25	—	—
Duration of single anxiety episode	—	4.80 ± 3.52	—	—
HAMA score	—	23.75 ± 5.67	—	—
GAF	—	61.50 ± 9.28	—	—
Medical				
Medication load index		1.55 ± 0.85		
Medications		No. patients		
SSRIs				
Fluoxetine		3		
Sertraline		6		
Paroxetine		15		
Citalopram		1		
Escitalopram		11		
Fluvoxamine		1		
SNRIs				
Venlafaxine		4		
Duloxetine		4		

*Note*: Values are mean ± *SD*.

Abbreviations: FD, framewise displacement; GAD, generalized anxiety disorder; HAMA, 14‐item Hamilton anxiety rating scale; HC, healthy controls; SNRIs, serotonin and norepinephrine reuptake inhibitors; SSRIs, selective serotonin reuptake inhibitors.

a Two‐sample *t* test (two‐tailed).

b Chi‐square *t* test.

### 2.2 Data acquisition

fMRI data for all subjects were acquired using a 3 T GE DISCOVERY MR750 scanner (General Electric, Fairfield Connecticut) equipped with an eight‐channel prototype quadrature birdcage head coil. Participants were instructed to keep their eyes closed, not think of anything, not fall asleep, and keep the head motionless during fMRI scanning. Resting‐state fMRI data were obtained using an echo‐planar imaging sequence with the following parameters: repetition time/echo time = 2,000/30 ms, matrix size = 64 × 64, field of view = 240 × 240 mm^2^, voxel size = 3.75 × 3.75 × 3.2 mm^3^, flip angle = 90°, 43 slices, no gap, and 255 volumes. The entire fMRI scan procedure lasted for 8 min and 30 s.

### 2.3 Data preprocessing

Resting‐state fMRI images were preprocessed using the Data Processing and Analysis of Brain Imaging (DPABI) toolbox (http://rfmri.org/dpabi). The preprocessing steps included: (a) discarding the first 15 volumes to stabilize the signal of the scanner and enable subjects to adapt to the environment; (b) slice timing correction for the remaining 240 fMRI images; (c) head motion correction (participants were excluded if their maximal head motion exceeded 3 mm displacement or 3° of rotation); (d) spatial normalization to standard Montreal Neurological Institute space and resampled to 3 × 3 × 3 mm^3^ resolution; (e) spatial smoothing using a Gaussian kernel with full‐width at half‐maximum of 6 mm; (f) detrending; (g) regression of nuisance covariates including the Friston‐24 motion parameters, white matter signals, cerebrospinal fluid signals, and global signal; (h) temporal band‐pass filtering at a frequency band of 0.01–0.08 Hz; (i) calculating the mean FD of each subject to evaluate the head movement (He et al., 2019; Lu et al., 2017); and (j) motion scrubbing to remove the “bad” time points and their 1‐back and 2‐forward time points on the basis of FD threshold of 0.5 mm (Power, Barnes, Snyder, Schlaggar, & Petersen, 2013).

### 2.4 dALFF analysis

The sliding window method was applied to evaluate the dALFF for each participant using the DynamicBC toolbox (Liao et al., 2014). Previous studies proposed that the window length is an open but essential parameter in sliding‐window‐based resting‐state dynamics computation (Li et al., 2018; Li, Duan, et al., 2019; Liao et al., 2019) To avoid the introduction of spurious fluctuations, the minimum window length should be larger than 1/*f*_min_, where *f*_min_ is the minimum frequency of time series (Leonardi & Van De Ville, 2015; Li, Wang, et al., 2018). Here, a window length of 50 TR was considered as the optimal parameter to maintain the balance between capturing a rapidly shifting dynamic relationship and obtaining reliable estimates of the correlations between regions (Li, Liao, et al., 2018; Li, Wang, et al., 2018; Pang et al., 2018). Hence, we selected 50 TR (100 s) as sliding‐window length and five TR (10 s) as step size to calculate the dALFF of each participant. The time series of each participant was divided into 39 windows, and the ALFF map was computed within each window, generating a set of ALFF maps for each participant. Subsequently, we measured the variance of these maps using *SD* to evaluate the temporal variability of dALFF (dALFF variability). Finally, for each participant, the dALFF variability of each voxel was further transformed into z‐scores by subtracting the mean and dividing by the *SD* of global values. Finally, for each participant, the dALFF variability of each voxel was further transformed into a z‐score by subtracting the mean and dividing by the *SD* of global values. The static ALFF (sALFF) map for each participant was obtained to verify whether dALFF and sALFF exhibited similar or complementary information to provide additional insight into the neuropathological mechanisms underlying GAD.

### 2.5 Statistics analysis

The dALFF variability value was averaged at each voxel across subjects within GAD and HC groups to obtain dALFF variability distribution in both groups. Two‐sample *t* test was performed to assess the group differences in dALFF variability between the GAD and HC groups, with age, sex, education level, and mean FD as covariates. Similarly, the sALFF distribution in the two groups was obtained by averaging sALFF values at each voxel across subjects of each group (GAD and HC). A two‐sample *t* test with the same covariates was applied to assess group differences in sALFF between patients with GAD and HCs. Multiple comparison correction was performed for two‐sample *t* tests using a voxel‐wise false discovery rate (FDR) approach, with a threshold of *p* < .05 and cluster size >20.

### 2.6 Multivariate pattern analysis

Multivariate pattern analysis (MVPA) is a useful classification approach that can identify the features contributing the most to the classification and classify patients at the individual level (Chen et al., 2016). In the present study, the region of interest (ROI)‐wise MVPA was used to test the abilities of abnormal dALFF and abnormal sALFF in classifying patients with GAD and HCs. In detail, two sets of ROIs were functionally defined by clusters with significant group differences in dALFF variability and sALFF, termed dALFF's ROIs and sALFF's ROIs, respectively. The mean dALFF variability values of each dALFF's ROIs and mean sALFF values of each sALFF's ROIs were extracted for each participant. Two classification analyses were conducted, in which the features were separately set to be (a) the dALFF variability values of dALFF's ROIs; and (b) the sALFF values of sALFF's ROIs. The leave‐one‐out cross‐validation (LOOCV) method was utilized to evaluate the performance of classifiers in both classification analyses, to produce a robust and reliable model. This method is proven to be an unbiased strategy and is suitable for small sample sizes (Finn et al., 2015; Shen et al., 2017). We assumed the presence of n samples in our study. In each LOOCV trial, *n* − 1 subjects' data were selected as the training set to train the classifying model, and the remaining subject's data was regarded as a test set to test the model. This procedure was repeated n times. The accuracy, sensitivity, specificity, and classification weights were reported for LOOCV analysis.

### 2.7 Medication information assessment

To test whether the dALFF variability of patients would be affected by medication, we calculated the medication load index of each patient to reflect the dosage of medication taken. The total medication load index was measured using an approach employed by previous studies (Almeida et al., 2009; Hassel et al., 2008; Versace et al., 2008) For each patient, we coded the dose of each medicine taken as 0 (absent), 1 (low), or 2 (high) based on previously developed criteria (Sackeim, 2001). Individuals on Levels 1 and 2 of these criteria were coded as low‐dose; those on Levels 3 and 4 were coded as high dose. A no‐dose subtype was added for patients who were not taking these medications. Two medications (escitalopram and duloxetine) that are not included in the criteria of Sackeim (2001) were coded as 0, 1, or 2 according to the midpoint of the daily dose range recommended by the Physician's Desk Reference. The total medication load for each patient was obtained by summing all medication codes.

### 2.8 Clinical correlation analysis

To further investigate the potential associations of abnormal dALFF variability and sALFF with symptom severity of patients with GAD, the mean dALFF variability values of each dALFF's ROI and mean sALFF values of each sALFF's ROI were extracted to calculate the Pearson's correlation coefficient with HAMA scores in GAD patients. In addition, Spearman's rank correlations between abnormal dALFF variability, abnormal sALFF, and total medication load index were also calculated to test the potential influence of medication treatment on the results. A statistically significant threshold of *p* < .05 (uncorrected) was set for all correlation analyses.

### 2.9 Validation analysis

To verify our findings of dALFF variability obtained from sliding‐window length of 50 TR, we performed auxiliary analyses with different sliding window lengths. We recalculated the main results by using two other window lengths (30 and 80 TR).

## 3 RESULTS

### 3.1 dALFF variability and sALFF results

**Figure 1:**
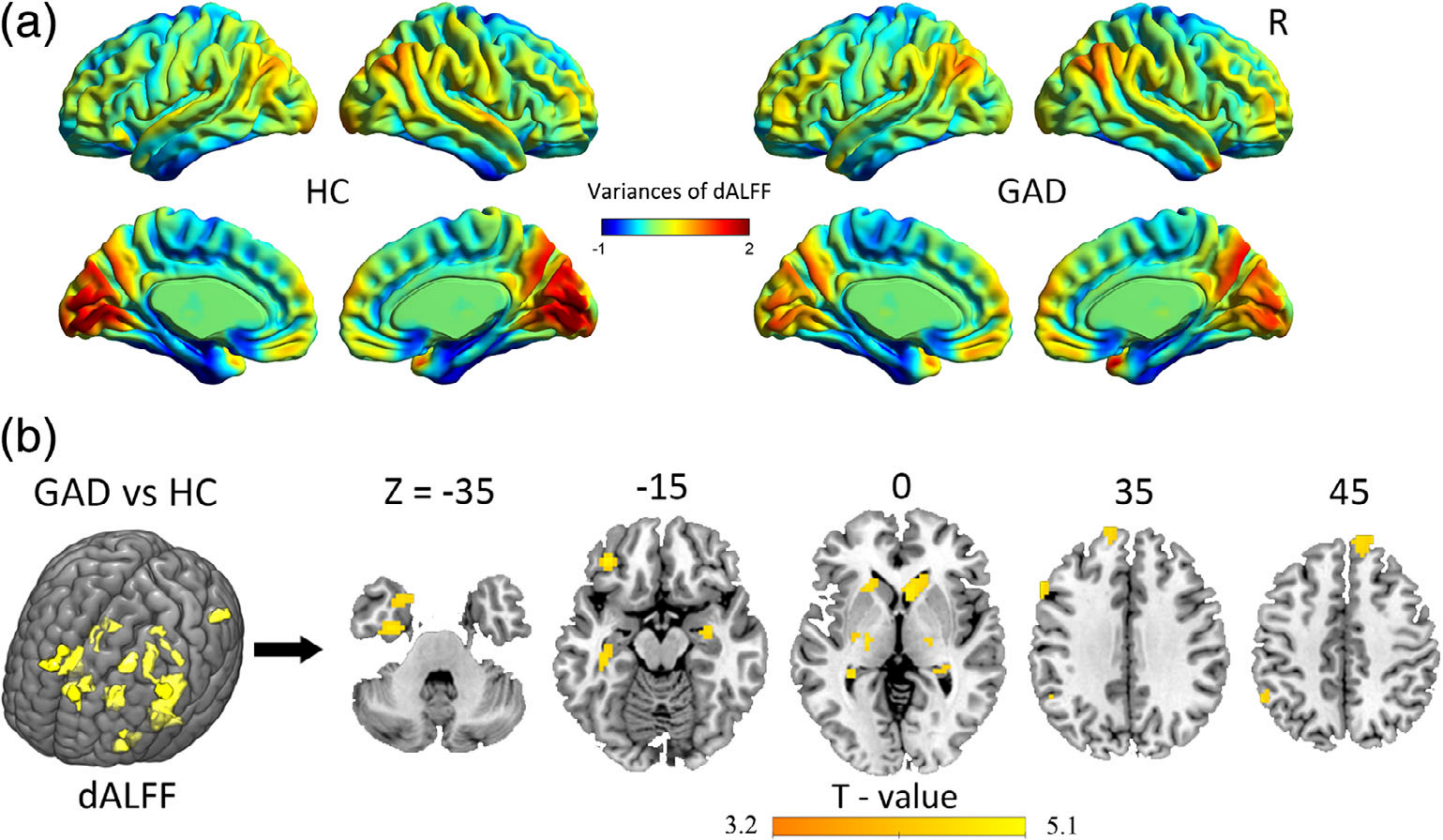
Pattern of dALFF variability in the HC and GAD groups (a) and brain regions with significant group differences in dALFF variability (b). Group differences in dALFF variability between the GAD and HC groups were identified using a two‐sample *t* test. The statistical significance level was set at *p* < .05, false discovery rate (FDR) corrected, *K* > 20. Patients with GAD showed increased dALFF variability in the bilateral hippocampus, thalamus, and striatum; and left OFC, IPL, TP, ITG, and fusiform. Abbreviations: ALFF, amplitude of low‐frequency fluctuation; dALFF, dynamic ALFF; HC, healthy control; GAD, generalized anxiety disorder; IPL, inferior parietal lobule; ITG, inferior temporal gyrus; L, left; OFC, orbital frontal cortex; R, right; TP, temporal pole

Patients with GAD and HC exhibited similar spatial distribution of dALFF variability, as shown in Figure 1. Brain regions with high dALFF variability were mainly located in the PFC, temporal–parietal junction, temporal pole, posteromedial, and occipital cortices. Brain regions with low variability were mainly located in the sensorimotor, inferior temporal, and limbic cortices. The group comparison results of dALFF variability showed that patients with GAD exhibited increased dALFF variability in the bilateral dorsomedial PFC (dmPFC), hippocampus, thalamus, and striatum; and left orbital frontal gyrus (OFC), inferior parietal lobule (IPL), temporal pole (TP), inferior temporal gyrus (ITG), and fusiform gyrus (*p* < .05, FDR corrected; Figure 1). The group comparison results of sALFF revealed that patients with GAD exhibited increased sALFF in the bilateral hippocampus, striatum, and left thalamus; and decreased sALFF in the bilateral postcentral and occipital cortices, and right fusiform (*p* < .05, FDR corrected; Figure S1).

### 3.2 Multivariate pattern analysis

**Figure 2:**
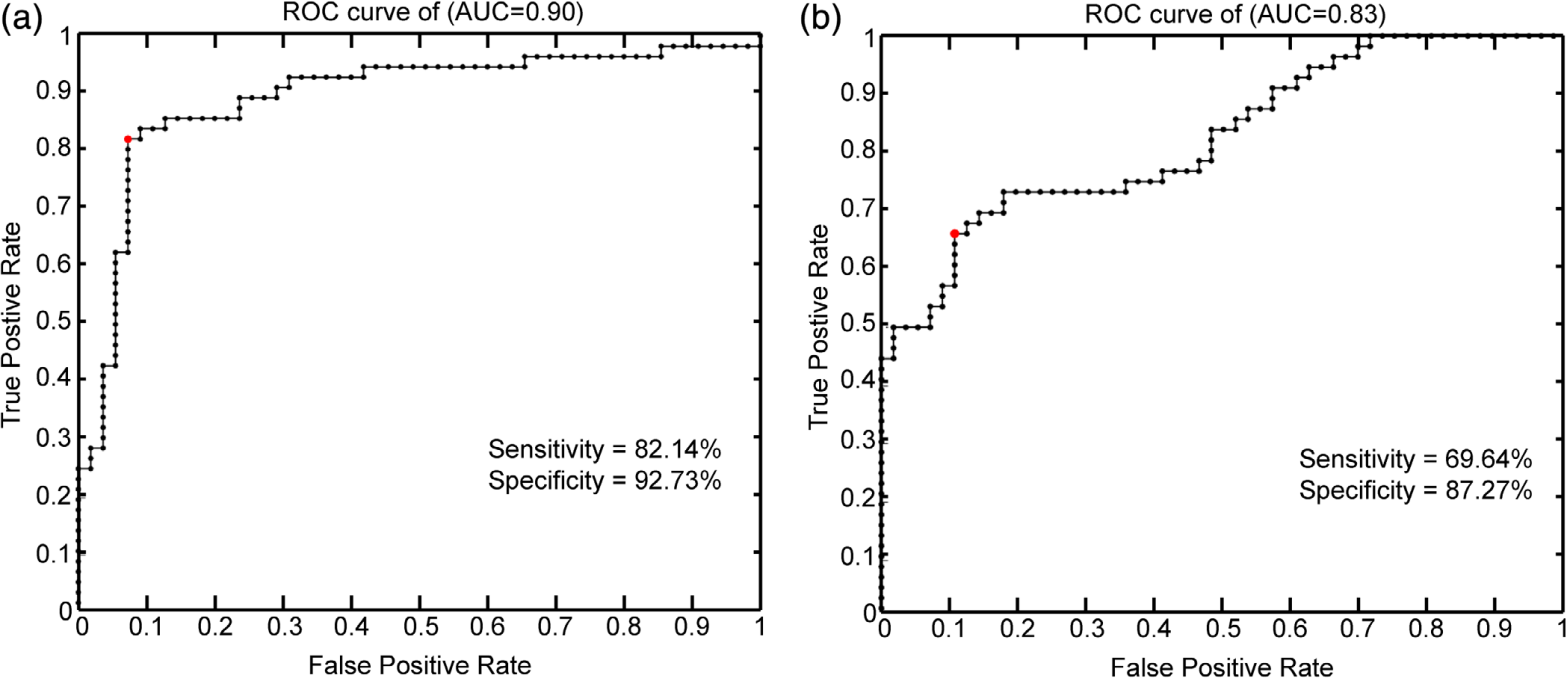
The results of classification analyses. The classification with altered dALFF variability as features achieved an accuracy of 87%, sensitivity of 82%, and specificity of 93% (a). The classification with altered static amplitude of low‐frequency fluctuation (sALFF) as features achieved an accuracy of 78%, sensitivity of 70%, and specificity of 87% (b)

The results of the two classification analyses are shown in Figure 2. Using abnormal dALFF variability as features, the classification analysis revealed that the dALFF variability values of the bilateral dmPFC and striatum, left TP and IPL, and right hippocampus contributed the most to differentiating patients with GAD from HCs, achieving an accuracy of 87%, sensitivity of 82%, and specificity of 93%. However, the highest accuracy achieved by the classification analysis using abnormal sALFF as features was 78%, with a sensitivity of 70% and specificity of 87%. These results indicated that abnormal dALFF variability may be more powerful than abnormal sALFF for distinguishing patients with GAD from HCs.

### 3.3 Correlation results

**Figure 3:**
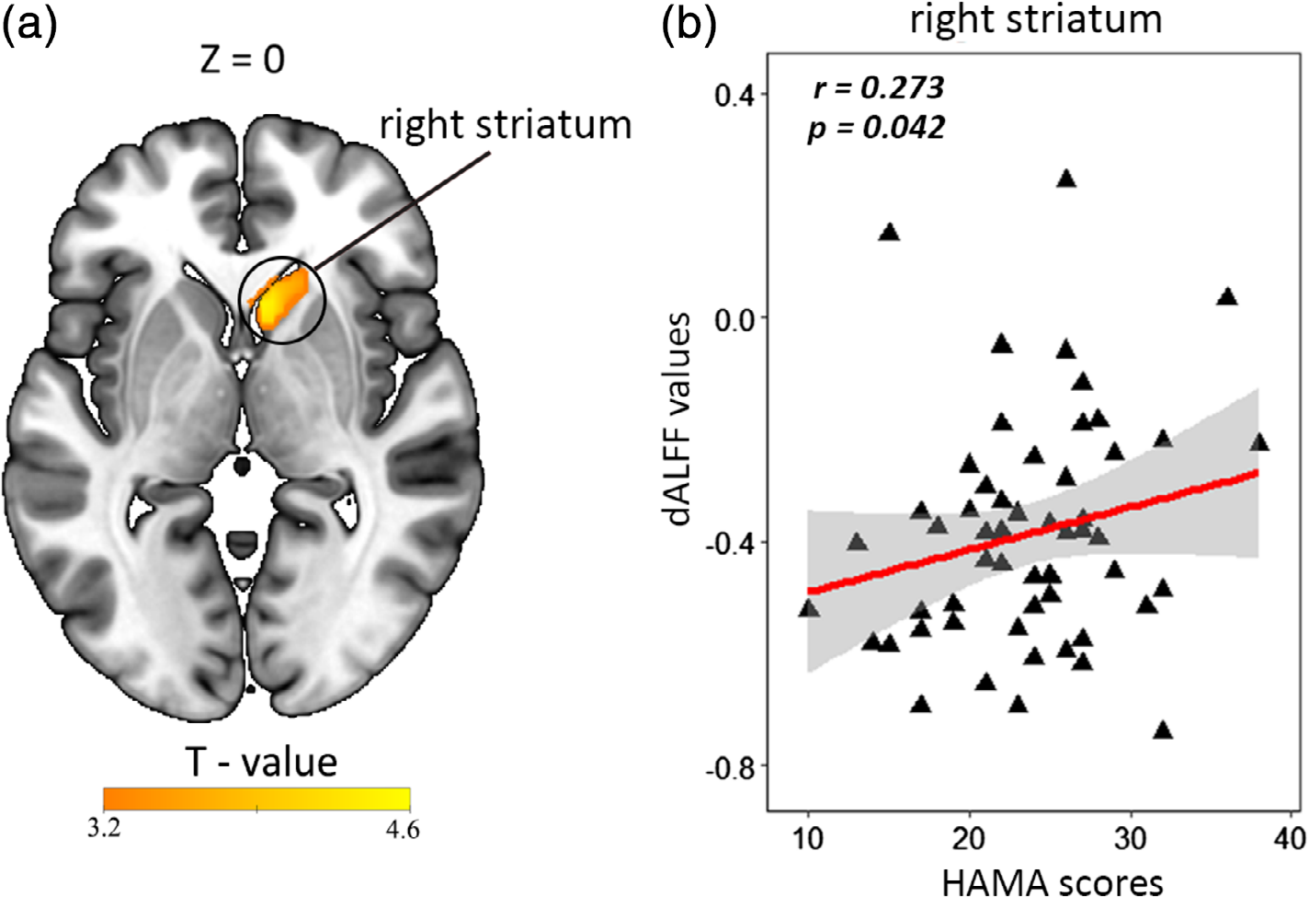
Dynamic amplitude of low‐frequency fluctuation (dALFF) variability in the right striatum was positively correlated with HAMA scores of patients with GAD; *r* = .273, *p* = .042

The abnormal dALFF variability of the right striatum was positively correlated with the HAMA scores of patients with GAD (*r* = .273, *p* = .042; Figure 3). However, the relationship between abnormal sALFF and HAMA scores in patients with GAD was not significant. No significant correlation was observed between either abnormal dALFF variability or abnormal sALFF and medication load index in GAD patients (for all correlation analyses, *p* > .1) (Tables S1 and S2), indicating that no significant medication effect on local brain activity was observed in this study.

### 3.4 Validation results

We validated our main results by using different sliding window lengths in this study. The findings of the sliding window length of 30 and 80 TRs were similar to the main results of 50 TR in our study. All validation analysis results are presented in Supplementary Materials (Figures S2 and S3).

## 4 DISCUSSION

This study is the first to investigate the temporal variability of local brain activity in GAD using a novel dALFF method. Patients with GAD exhibited increased dALFF variability in the hippocampus, thalamus, striatum, fusiform, and widespread prefrontal and parietal cortices. The dALFF variability in these regions could be used to classify patients with GAD and HCs, achieving an accuracy of 87%. The classification accuracy was superior when using dALFF variability compared to that using sALFF as features. Additionally, the abnormal dALFF variability in the striatum was correlated with symptom severity of GAD. These findings highlight the importance of considering dynamic local brain activity in GAD.

GAD showed increased dALFF variability in the hippocampus and thalamus, limbic regions which are involved in emotion processing. Of note, emotional dysfunction is the most prominent feature of GAD (Mochcoyitch, da Rocha Freire, Garcia, & Nardi, 2014; Via et al., 2018), such as reduced capacity for engaging emotion‐regulation brain networks when viewing and adjusting feelings toward positive and negative affective pictures (Blair et al., 2012), attenuated blood oxygen level‐dependent response of the PFC and ACC to emotional expressions (Palm et al., 2011), and inability to adapt to emotional conflict (Etkin, Prater, Hoeft, Menon, & Schatzberg, 2010). The hippocampus is involved in the ventral affective neural systems (Moon & Jeong, 2015) and plays a critical role in mediating anxiety states in coordination with other limbic regions (Caliskan & Stork, 2018). GAD has been reported to exhibit hyperactivation in the hippocampus during processing anxiety‐inducing distractors (Moon & Jeong, 2015; Park et al., 2016). Such hyperactivation was correlated with pathological anxiety responses and considered to be associated with emotional dysregulation in GAD (Moon & Jeong, 2015). The thalamus is a relay center for sensory information transmission (Sherman, 2007). It has connections with widespread cortical and subcortical regions (Qiao et al., 2017) and participates in multiple cognitive and emotional processes (Jiang et al., 2018). Previous studies reported hyperactivation of the thalamus in GAD during imagining disorder‐related scenarios (Buff et al., 2018), and increased rsFCs of the thalamus in GAD patients (Etkin et al., 2009; Qiao et al., 2017). Consistent with functional impairments, GAD also exhibits structural abnormalities in the limbic system, such as reduced gray matter volume in regions including the hippocampus, thalamus and insula (Abdallah et al., 2013; Moon, Kim, & Jeong, 2014; Moon, Yang, & Jeong, 2015). Our findings of increased dALFF in limbic regions are consistent with previous findings that patients with GAD show overhyperactivation in the limbic system, which may underscore the excessive sensitivity to emotional stimuli, especially those of negative valence, a typical clinical manifestation of GAD (Novick‐Kline, Turk, Mennin, Hoyt, & Gallagher, 2005).

Increased dALFF variability was also observed in the dmPFC and IPL, components of the frontoparietal cognitive control network (Niendam et al., 2012). Several studies have highlighted the important role of emotional dysregulation in the development and maintenance of GAD (Blair et al., 2012; Etkin et al., 2010; Mochcoyitch et al., 2014; Wang et al., 2018). Previous neuroimaging studies have demonstrated that the difficulty of emotion regulation in GAD may result from the failure to recruit prefrontoparietal networks to downregulate emotional responses (Wang et al., 2018). GAD is underscored by elevated static regional neural activity in the dmPFC and aberrant rsFC of the dmPFC with limbic regions, temporal lobe, vmPFC, and precentral gyrus (Wang, Hou, et al., 2016). Enlarged gray matter volume (GMV) (Schienle, Ebner, & Schaefer, 2011) and weakened emotion regulation‐related activity of the dmPFC have been also reported in GAD (Etkin et al., 2010; Wang et al., 2018). Additionally, the IPL was hypoactivated during cognitive reappraisal of emotion tasks in patients with several subtypes of anxiety disorders (Wang et al., 2018) and exhibited decreased cortical thickness in GAD (Abdallah et al., 2012). The increased dALFF variability in dmPFC and IPL observed in the current study indicates aberrant temporal fluctuations of local brain activity in these regions. Such abnormal patterns may disrupt the capacity to engage the prefrontoparietal network in emotion regulation in GAD.

Regions previously involved in high‐level socioemotional function, including the OFC, striatum, fusiform gyrus, TP, and ITG, also exhibited increased dALFF variability in GAD. As key nodes of the reward system, the OFC and striatum are strongly engaged in reward processing. Impairments of the brain reward system are evident in GAD, including reduced OFC GMV (Strawn et al., 2013) and disrupted functional connectivity of the striatum (Qiao et al., 2017). The fusiform gyrus is involved in facial recognition (Park et al., 2016) and affective stimuli perception (Geday, Gjedde, Boldsen, & Kupers, 2003), and the TP and ITG are implicated in theory of mind (TOM). These regions play crucial roles in the perception and reasoning of social cues, such as other persons' beliefs and emotions, which are essential for social functioning (Cui et al., 2017; Kohn et al., 2014). The abnormal dALFF detected in these regions is consistent with previous studies suggesting that GAD patients exhibit impaired TOM reasoning for negative social stimuli (Kohn et al., 2014), with structural and functional abnormalities in regions associated with social cognition, such as abnormal rsFC of the fusiform gyrus with limbic regions (Cui et al., 2016; Molent et al., 2018) and aberrant cortical morphology and white matter integrity of the ITG and TP (Hilbert et al., 2015; Molent et al., 2018; Strawn et al., 2013).

Static and dALFF revealed similar group differences. However, dALFF contributed more than sALFF to distinguishing between patients with GAD and HCs. With dALFF variability values as features, classification achieved a relatively high accuracy of 87%. In addition, increased dALFF variability in the striatum was positively correlated with symptom severity, suggesting that the increased dALFF variability in this brain region may be important for understanding the development of anxiety. These findings suggest that dynamic local brain activity may be a powerful neuroimaging indicator for probing pathological changes in GAD and provide a new avenue to distinguish patients from the healthy population.

This study has several limitations. First, the selection of the sliding window length remains a topic of debate, and optimal length for obtaining the dynamics of brain activity is unclear. We selected 50 TR as window length on the basis of the criteria that the minimum length should be larger than 1/*f*_min_, which was proposed by previous studies (Leonardi & Van De Ville, 2015; Li, Duan, et al., 2019). The results of different sliding window lengths were similar to the main results of 50 TR, demonstrating that our findings of dALFF variability were relatively stable. Second, given the high comorbidity of anxiety and depression, excluding individuals with depressive disorder may decrease the generalizability of our findings. More comorbid samples are required to replicate and complement our findings. Future studies will benefit from the systematic investigation of common and distinct neural mechanisms underlying anxiety and anxiety comorbidity in other affective disorders to obtain deeper understanding of the neural mechanisms underlying GAD. Third, most of our patients took medications, which may affect the reliability of our results. Although the correlations between abnormal dALFF variability and medication load index were not significant, our results should be confirmed by future studies with medication‐naive patients.

## 5 CONCLUSION

In summary, patients with GAD exhibited increased temporal variability of dALFF in regions implicated in executive, emotional, and social function. The abnormal dALFF variability was correlated with symptomatology of GAD and contributed to distinguishing patients with GAD from HCs with higher accuracy than that achieved using abnormal sALFF as features. This study sheds new insight into the brain dysfunction underlying GAD from the perspective of dynamic local brain activity, highlighting the important role of alterations in dALFF variability in understanding the neuropathological mechanisms underscoring GAD and potentially informing the diagnosis of this disease.

## CONFLICT OF INTEREST

The authors declare no potential conflict of interest.

## Supporting information


**Table S1** Correlations between medication load and dALFF variability value of regions with significant group differences between patients with GAD and HCs.
**Table S2**. Correlations between total medication load and sALFF value of regions with significant group differences between patients with GAD and HCs.
**Figure S1**. Pattern of sALFF in the HC and GAD groups (A) and brain regions with significant group difference in sALFF (B). Group differences in sALFF between the GAD and HC groups were identified using a two‐sample *t*‐test. The statistical significance level was set as *p* < 0.05, FDR corrected, *K > 20*. Patients with GAD showed increased sALFF in the bilateral hippocampus, striatum, left thalamus; and decreased sALFF in the bilateral postcentral occipital cortices and the right fusiform gyrus. Abbreviations: HC, healthy control; GAD, generalized anxiety disorder; L, left; R, right.
**Figure S2**. Brain regions with significant group differences in dALFF variability (30 TR). dALFF variability between the GAD and HC groups was identified using a two‐sample *t*‐test. The statistical significance level was set at *p* < 0.05, FDR‐corrected, *K > 20*. Patients with GAD showed increased dALFF varialbity in the bilateral dmPFC, hippocampus, thalamus, and striatum; left OFC, IPL, TP, ITG, and fusiform gyrus; and decreased dALFF variability in the bilateral occipital cortices. Abbreviations: HC, healthy control; GAD, generalized anxiety disorder; dmPFC, dorsal medial prefrontal cortex; OFC, orbital frontal cortex; IPL, inferior parietal lobule; TP, temporal pole; ITG, inferior temporal gyrus.
**Figure S3**. Brain regions with significant group differences in dALFF variability (80 TR). dALFF variability between the GAD and HC groups was identified using a two‐sample *t*‐test. The statistical significance level was set as *p < 0.001*, uncorrected. Patients with GAD showed increased dALFF variability in the bilateral dmPFC, hippocampus, and striatum; left OFC, IPL, TP, ITG and fusiform gyrus; and decreased dALFF variability in the bilateral occipital cortices. Abbreviations: HC, healthy control; GAD, generalized anxiety disorder; dmPFC, dorsal medial prefrontal cortex; OFC, orbital frontal cortex; IPL, inferior parietal lobule; TP, temporal pole; ITG, inferior temporal gyrus.

## Data Availability

Data sharing is not applicable to this article as no new data were created or analyzed in this study.
