# Supplementary material for: Dynamic changes of amplitude of low‐frequency fluctuations in patients with generalized anxiety disorder
Source: Hum Brain Mapp. 2019 Dec 17;41(6):1667–76. doi: 10.1002/hbm.24902 (PMC7267950; doi:10.1002/hbm.24902)
Supplement: Supplementary file 1 — Table S1 Correlations between medication load and dALFF variability value of regions with significant group differences between patients with GAD and HCs. Table S2. Correlations between total medication load and sALFF value of regions with significant group differences between patients with GAD and HCs. Figure S1. Pattern of sALFF in the HC and GAD groups (A) and brain regions with significant group difference in sALFF (B). Group differences in sALFF between the GAD and HC groups were identified using a two‐sample t‐test. The statistical significance level was set as p < 0.05, FDR corrected, K > 20. Patients with GAD showed increased sALFF in the bilateral hippocampus, striatum, left thalamus; and decreased sALFF in the bilateral postcentral occipital cortices and the right fusiform gyrus. Abbreviations: HC, healthy control; GAD, generalized anxiety disorder; L, left; R, right. Figure S2. Brain regions with significant group differences in dALFF variability (30 TR). dALFF variability between the GAD and HC groups was identified using a two‐sample t‐test. The statistical significance level was set at p < 0.05, FDR‐corrected, K > 20. Patients with GAD showed increased dALFF varialbity in the bilateral dmPFC, hippocampus, thalamus, and striatum; left OFC, IPL, TP, ITG, and fusiform gyrus; and decreased dALFF variability in the bilateral occipital cortices. Abbreviations: HC, healthy control; GAD, generalized anxiety disorder; dmPFC, dorsal medial prefrontal cortex; OFC, orbital frontal cortex; IPL, inferior parietal lobule; TP, temporal pole; ITG, inferior temporal gyrus. Figure S3. Brain regions with significant group differences in dALFF variability (80 TR). dALFF variability between the GAD and HC groups was identified using a two‐sample t‐test. The statistical significance level was set as p < 0.001, uncorrected. Patients with GAD showed increased dALFF variability in the bilateral dmPFC, hippocampus, and striatum; left OFC, IPL, TP, ITG and fusiform gyrus; and dec [file HBM-41-1667-s001.docx]

**Supplementary materials for**

“Dynamic changes of amplitude of low frequency fluctuations in patients with generalized anxiety disorder”

Qian Cui^1^, Wei Sheng^2^, Yuyan Chen^2^, Yajing Pang^2^, Fengmei Lu^2^, Qin Tang^2^, Shaoqiang Han^2^, Qian Shen^3^, Yifeng Wang^2^, Ailing Xie^1^, Jing Huang^2^, Di Li^2^, Ting Lei^1^, Zongling He^2^, Huafu Chen^2^*

^1^School of Public Affairs and Administration, University of Electronic Science and Technology of China, Chengdu, China

^2^The Clinical Hospital of Chengdu Brain Science Institute, MOE Key Lab for Neuroinformation, School of life Science and technology, University of Electronic Science and Technology of China, Chengdu, China

^3^Education Center for Students Cultural Qualities, University of Electronic Science and Technology of China, Chengdu, China

***Corresponding authors:**

**Huafu Chen:** [chenhf@uestc.edu.cn](mailto:chenhf@uestc.edu.cn)

School of Life Science and Technology, Center for Information in Medicine, University of Electronic Science and Technology of China, Chengdu, China

**Table S1**. Correlations between medication load and dALFF variability value of regions with significant group differences between patients with GAD and HCs.

| **Regions** |  | **Total medication load** |
| --- | --- | --- |
|  |  | **Spearman’s *rho (p)*** |
| **GAD >HC** |  |  |
| Left dmPFC |  | -0.149 (0.274) |
| Right dmPFC |  | -0.034 (0.803) |
| Left hippocampus |  | -0.025 (0.856) |
| Right hippocampus |  | -0.129 (0.344) |
| Left thalamus |  | 0.071 (0.603) |
| Right thalamus |  | -0.094 (0.493) |
| Left striatum |  | -0.057 (0.676) |
| Right striatum |  | 0.065 (0.636) |
| Left OFC |  | -0.078 (0.568) |
| Left IPL |  | -0.077 (0.569) |
| Left TP |  | -0.180 (0.185) |
| Left ITG |  | -0.119 (0.383) |
| Left fusiform gyrus |  | -0.001 (0.996) |

**Abbreviations:** dALFF, dynamic amplitude of low-frequency fluctuation; GAD, generalized anxiety disorder; HC, healthy control; dmPFC, dorsalmedial prefrontal cortex; OFC, orbital frontal cortex; IPL, inferior parietal cortex; TP, temporal pole; ITG, inferior temporal gyrus.

**Table S2.** Correlations between total medication load and sALFF value of regions with significant group differences between patients with GAD and HCs.

| **Regions** |  | **Total medication load** |
| --- | --- | --- |
|  |  | **Spearman’s *rho (p)*** |
| **GAD > HC** |  |  |
| Left hippocampus |  | 0.221 (0.118) |
| Right hippocampus |  | 0.049 (0.723) |
| Left striatum |  | -0.005 (0.969) |
| Right striatum |  | 0.065 (0.633) |
| Left thalamus |  | -0.084 (0.536) |
| **GAD < HC** |  | -0.094 (0.493) |
| Right fusiform gyrus |  | 0.016 (0.905) |
| Left occipital cortex |  | 0.025 (0.852) |
| Right calcarine |  | 0.060 (0.662) |
| Left postcentral gyrus |  | -0.042 (0.760) |
| Right postcentral gyrus |  | 0.020 (0.883) |

**Abbreviations:** sALFF, static amplitude of low-frequency fluctuation; GAD, generalized anxiety disorder; HC, healthy control.


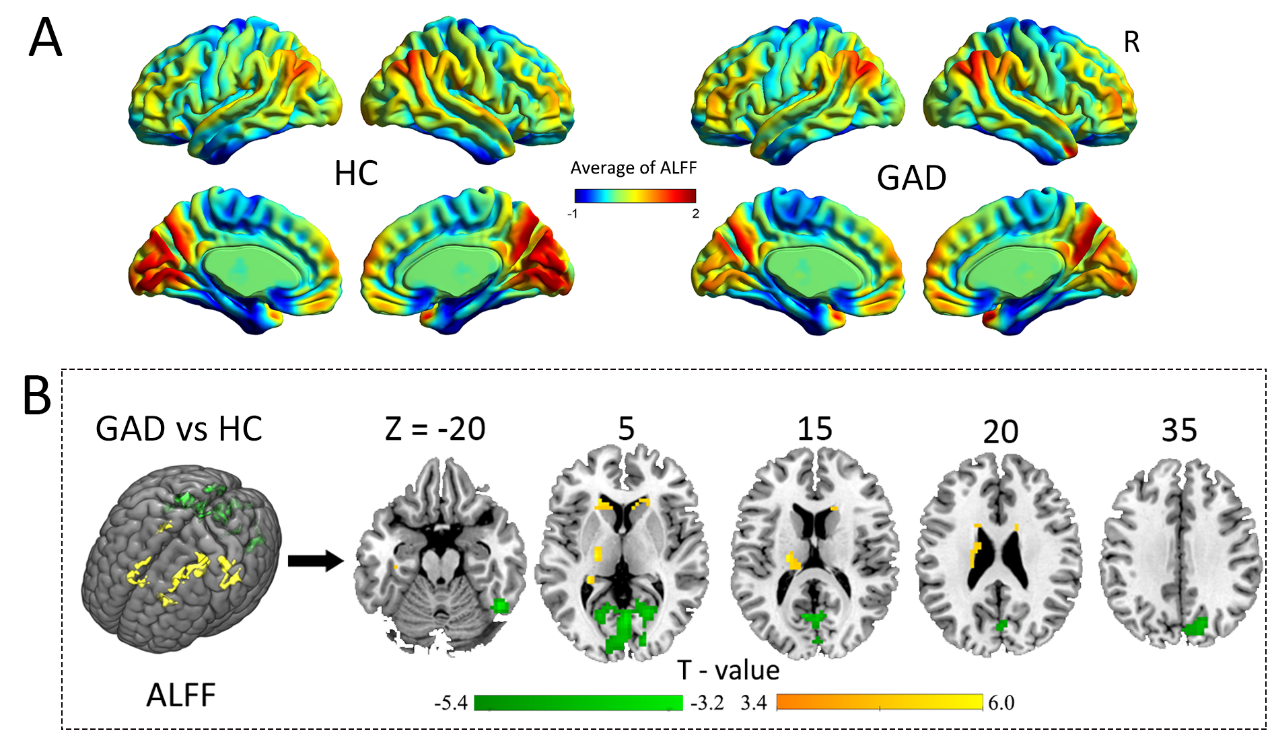


**Figure S1.** Pattern of sALFF in the HC and GAD groups (A) and brain regions with significant group difference in sALFF (B). Group differences in sALFF between the GAD and HC groups were identified using a two-sample *t*-test. The statistical significance level was set as *p* <0.05, FDR corrected, *K >20*. Patients with GAD showed increased sALFF in the bilateral hippocampus, striatum, left thalamus; and decreased sALFF in the bilateral postcentral occipital cortices and the right fusiform gyrus. Abbreviations: HC, healthy control; GAD, generalized anxiety disorder; L, left; R, right.


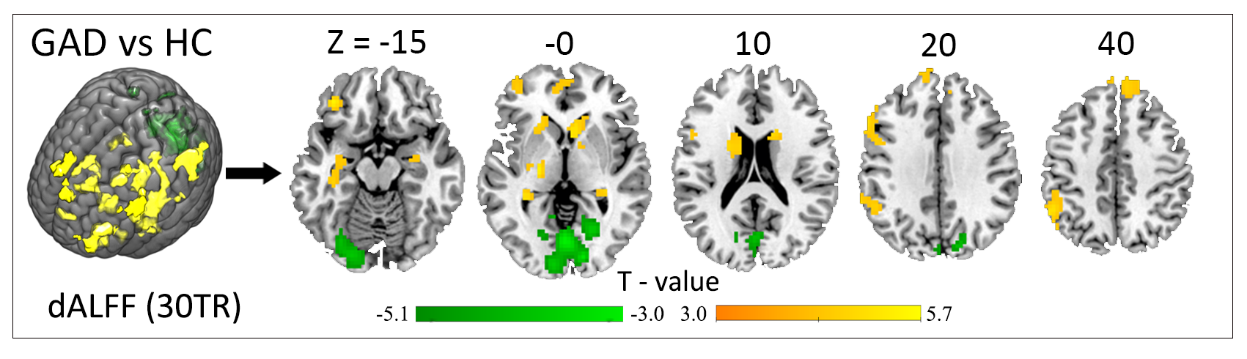


**Figure S2**. Brain regions with significant group differences in dALFF variability (30 TR). dALFF variability between the GAD and HC groups was identified using a two-sample *t*-test. The statistical significance level was set at *p* <0.05, FDR-corrected*, K >20*. Patients with GAD showed increased dALFF varialbity in the bilateral dmPFC, hippocampus, thalamus, and striatum; left OFC, IPL, TP, ITG, and fusiform gyrus; and decreased dALFF variability in the bilateral occipital cortices. Abbreviations: HC, healthy control; GAD, generalized anxiety disorder; dmPFC, dorsal medial prefrontal cortex; OFC, orbital frontal cortex; IPL, inferior parietal lobule; TP, temporal pole; ITG, inferior temporal gyrus.


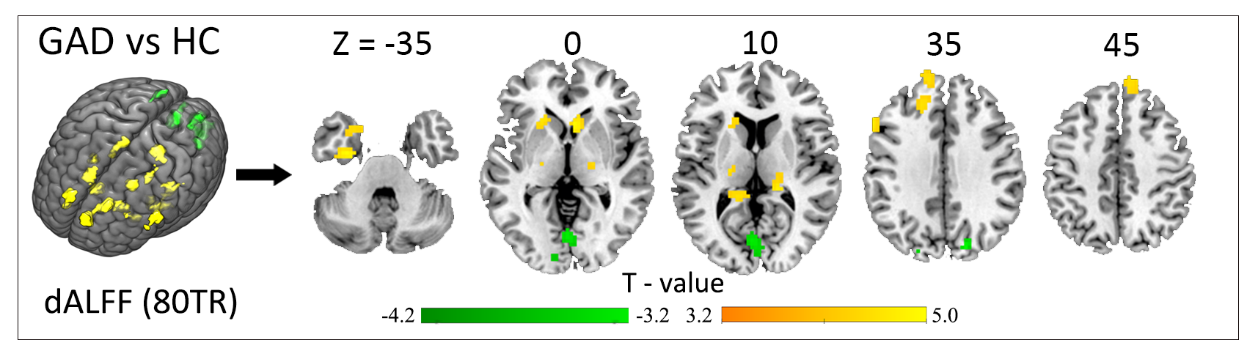


**Figure S3**. Brain regions with significant group differences in dALFF variability (80 TR). dALFF variability between the GAD and HC groups was identified using a two-sample *t*-test. The statistical significance level was set as *P <0.001*, uncorrected. Patients with GAD showed increased dALFF variability in the bilateral dmPFC, hippocampus, and striatum; left OFC, IPL, TP, ITG and fusiform gyrus; and decreased dALFF variability in the bilateral occipital cortices. Abbreviations: HC, healthy control; GAD, generalized anxiety disorder; dmPFC, dorsal medial prefrontal cortex; OFC, orbital frontal cortex; IPL, inferior parietal lobule; TP, temporal pole; ITG, inferior temporal gyrus.
